# Targeted Activation of T Cells with IL-2-Coupled Nanoparticles

**DOI:** 10.3390/cells9092063

**Published:** 2020-09-09

**Authors:** Verena K. Raker, Christian Becker, Katharina Landfester, Kerstin Steinbrink

**Affiliations:** 1 Department of Dermatology, Johannes Gutenberg-University Mainz, 55122 Mainz, Germany; rakerv@uni-mainz.de (V.K.R.); christian.becker@unimedizin-mainz.de (C.B.); 2 Max Planck Institute for Polymer Research, 55128 Mainz, Germany; landfest@mpip-mainz.mpg.de; 3 Department of Dermatology, Westfälische Wilhelms-University Münster, 48149 Münster, Germany

**Keywords:** interleukin-2, nanoparticles, immunotherapy

## Abstract

Interleukin-2 (IL-2) is a T cell growth factor particularly required in regulatory T cell maintenance and memory T cell responses. High-dose IL-2 treatment was the first FDA-approved immunotherapy for cancer, while low-dose IL-2 administration has shown promise in allograft rejection and autoimmune and inflammatory diseases. However, its pleiotropic nature and the existence of IL-2 receptors with different binding affinity limit its therapeutic application. For an improved clinical applicability of the cytokine, a targeted receptor assignment must, therefore, be achieved. Nanoparticles allow controlling the location and dose of immunomodulating compounds and to specifically address specific receptors through targeted drug binding. In this review article we discuss the IL-2 biology and current clinical application with regard to nanoparticle-based IL-2-mediated manipulation of T cell responses in autoimmunity, chronic inflammation, and cancer.

## 1. Introduction

The use of nanoparticles for the administration of immunotherapeutics is advantageous compared to the use of free drugs, as all parameters of the effect (site of action, release, whereabouts) can be precisely controlled. Nanoparticles can, for example, protect their cargo from the surrounding biological milieu, extend its half-life, minimize its systemic toxicity, and control its delivery to defined immune or tumor cells. Due to these possibilities, nanoparticles will become an effective treatment option for allergic and autoimmune diseases as well as for cancer therapy [1,2,3,4]. Interleukin-2 (IL-2) is a T-cell growth factor, which became one of the first FDA-approved immunotherapeutic agents for the treatment of metastatic melanoma and renal cell cancer [5,6]. While its use in cancer therapy was high-dose, it was subsequently recognized that low doses of IL-2 preferentially expand tolerance-inducing regulatory T cells. Due to this effect, IL-2 is now successfully used in low doses to suppress graft rejection, autoimmune reactions, and chronic inflammation [7,8]. The context and dose-dependent different effects of IL-2 are based on the different distribution of differently affine monomeric, dimeric, and trimeric IL-2 receptors within the immune system [9]. While naïve CD8^+^ T cells, CD4^+^/CD8^+^ memory T cells, and NK (T) cells express the dimeric receptor with low affinity, regulatory T cells constitutively express the trimeric IL-2R with high affinity. In order to control the effect of therapeutically administered IL-2, attempts are being made to target it to low- or high-affinity receptors and to extend its half-life. Nanoparticle-based approaches combine these strategies and at the same time offer new possibilities to make IL-2 therapeutically more useful.

## 2. IL-2 and Its Initial Use in Cancer Therapy 

IL-2 is the second identified immune cell growth and differentiation factor (cytokine) and the first that has been cloned and recombinantly produced [10,11]. Due to its originally described effect as an autocrine survival and proliferation signal for T cells and the indispensable role of T cells in anti-tumor immunity [12], IL-2 was explored in cancer therapy early after its discovery [13]. Based on its dose-dependent effect on T cell proliferation and notably short half-life (<30 min) in circulation [14], IL-2 was first administered in escalating doses of up to 120 million IU (MIU) in bolus infusions every eight hours. The maximum tolerated dose (126–150 MIU per day) achieved permanent tumor responses in a small subgroup of patients with melanoma and renal cell carcinoma [15], but was accompanied by massive cytokine release, inflammatory reaction, and vascular leak syndrome consisting of increased loss of intravascular fluid into extravascular spaces caused by interaction of IL-2 with IL-2 receptors on endothelial cells [16], reducing cell adhesion of the endothelia to each other or to the extracellular matrix [17]. Attempts to avoid the side effects by reducing the dose (to 72,000 U/kg) resulted in a loss of effect on tumor growth [18]. Despite its low safety profile, IL-2 became an FDA-approved drug (Proleukin, Novartis; generic name Aldesleukin) for renal cell carcinoma and metastatic melanoma treatment.

### 2.1. Producing and Responding Cells

Cell-specific IL-2 knockouts and a fluorescent reporter mouse strain revealed that T cells are the main IL-2 source, although B cells and dendritic cells (DCs) contribute in select organs [19,20,21,22]. In T cells, IL-2 is rapidly and transiently produced upon engaging the T cell receptor (TCR) and costimulation by molecules such as CD28 on naive T cells. The transient nature of IL-2 secretion depends on transcriptional induction by TCR signals and stabilization of IL-2 mRNA by costimulatory signals, followed by transcriptional silencing of the IL-2 gene and rapid degradation of IL-2 mRNA [23]. One of the two IL-2 genes is randomly selected and transcribed first during T-cell activation, although IL-2 expression can become bi-allelic. However, since IL-2 transcripts are rapidly degraded IL-2 production appears monoallelic [22].

**Figure 1:**
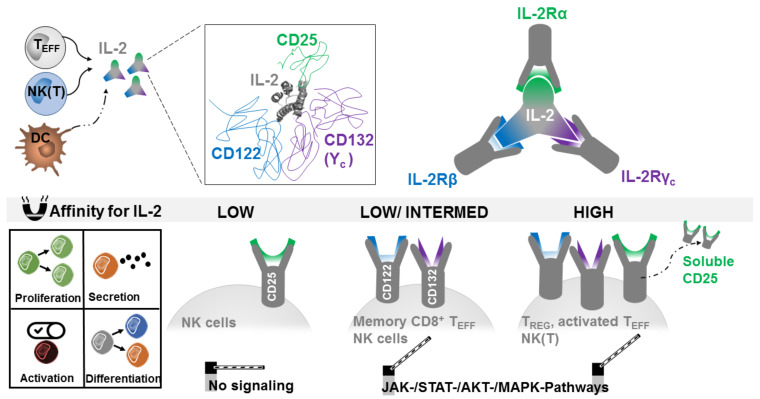
Interleukin-2 (IL-2) production, epitopes, and receptors. Interleukin-2 produced by T effector cells (T_EFF_), natural killer (T) cells (NK(T)), and dendritic cells (DC) binds to IL-2 receptors (IL-2Rs) CD25, CD122, and CD132. CD25 can be cleaved from the surface as a soluble form. Coexpression of CD122 and CD132 found on CD8 memory T cells has a low to intermediate IL-2 affinity, whereas the expression of all three receptors on activated T cells as well as regulatory T cells has high IL-2 affinity. IL-2-signaling pathways utilize different transcription factors and lead to proliferation, secretion of cytokines, activation, and differentiation of T cells.

While the reasons for occasional remissions in IL-2-treated cancer patients remained unclear, further investigations continued to clarify its mode of action. Three different IL-2 receptor chains termed alpha (CD25), beta (CD122), and gamma (CD132, also known as the common cytokine receptor γ chain) could be identified (Figure 1) [24]. CD25 binds IL-2 with low affinity (K_d_ ~ 10^−8^ M), CD122 and CD132 form a receptor of intermediate affinity (K_d_ ~ 10^−9^ M) and the combination of all three receptor chains generates an IL-2 receptor with high affinity (K_d_ ~ 10^−11^ M). CD122 and CD132 contain larger cytoplasmic domains whose heterodimerization is required for IL-2 signaling. In contrast, CD25 contains only a very short cytoplasmic domain and probably serves mainly to regulate the receptor affinity for IL-2. However, activated T cells also appear to secrete a soluble form of CD25 (sCD25) with currently unclear significance.

IL-2 binding to its receptors initiates signal transduction for the transcription of target genes through multiple signaling pathways. These include the Janus kinase (JAK) signal transducer and activator of transcription (STAT) pathway, the phosphoinositide 3-kinase (PI3K) Akt pathway, and the mitogen-activated protein kinase (MAPK) pathway (Figure 1) [25]. All of these three major pathways mediate the effect of IL-2 on cell proliferation, activation, differentiation, survival, and cytokine production in the immune cells. IL-2 presented bound to cellular IL-2R could also act in trans [26]. However, it should be noted that the affinity with which IL-2 can bind to IL-2R is relatively low with rapid on and off rates.

Contrary to the postulated role of IL-2 as a general T cell growth factor, knockout mice lacking the IL-2 gene as well as mice lacking CD122 or CD25 receptor chains did not show immunodeficiency but autoimmunity [27,28,29]. Subsequently, it was discovered that a small population of regulatory T cells (Treg), which is of central importance for the maintenance of peripheral immune tolerance, constitutively expresses CD25 and high affinity IL-2 receptors and is unable to produce IL-2 [30,31]. Further studies revealed that IL-2 acts as a key Treg survival and maintenance factor, in part through epigenetic changes in the transcription factor FOXP3 gene, which determines their function [32]. The importance of IL-2 in maintaining Treg was further confirmed by their absence in IL-2 deficiency and by the fact that adoptively transferred Treg suppresses the development of autoimmunity in CD122-deficient mice [33]. One common observation of studies in IL-2^−/−^, CD25^−/−^, or CD122^−/−^ mice is that there is substantial T cell proliferation [25,34,35]. The conclusion from these observations is that IL-2 is less necessary for the development of effector T cells than for the maintenance of Treg, indispensable for immune tolerance and homeostasis.

**Figure 2:**
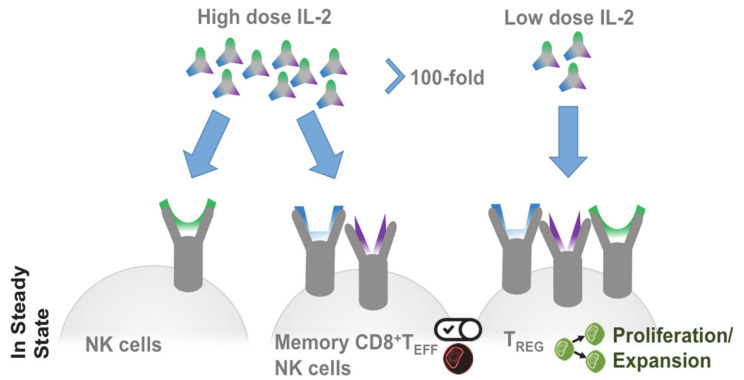
IL-2 dose-dependent receptor response. In steady state low to intermediate receptors respond to high doses of IL-2. A 100-fold reduction of the IL-2 dose targets regulatory T cells through their high affinity IL-2 receptor and leads to selective proliferation and expansion of regulatory T cells (Treg).

The effects of IL-2 can be explained by the distribution of its receptors (Figure 2): At steady state high-affinity IL-2 receptors are preferentially expressed on Treg, while their expression is induced by activation on other immune cells. These include, in particular, T cells, NK cells, and NK T cells, which are also the primary IL-2 source besides dendritic cells [36]. At low IL-2 levels, this receptor distribution prefers Treg, while at high concentrations the cytokine also regulates cells with low affinity receptors, such as NK cells [37]. Improved knowledge about IL-2 and IL-2 receptors led to clinical studies investigating the possibility to therapeutically increase Treg numbers in autoimmune and inflammatory patients by low amounts of IL-2 [38,39]. Studies with low-dose IL-2 used different doses (in the range of 0.33 and 3 MIU per day) and different timelines. However, robust-dose and time-dependent Treg expansion was achieved in all studies and at all doses [40,41]. Depending on the treated disease and its course, adapted regimens have been developed that use mostly symptom-free phases to propagate Treg. Remarkably, in all low-dose IL-2 studies in autoimmune diseases, the expansion of Treg does not lead to generalized immunosuppression but restores immunoregulatory mechanisms [7]. Moreover, in contrast to high doses, low doses of IL-2 are well tolerated [38,42].

### 2.2. Treg in Cancer

Increased Treg frequencies in tumor tissue are associated with a poor prognosis in a large number of tumors [43,44,45] and, experimentally, immune responses to transplanted tumors improve after selective Treg removal [46,47,48]. These observations have led to the widespread idea that Treg helps the tumor to escape an efficient antitumor immune response [49]. However, there are also observations to the contrary: Increased Treg cell counts in colon and breast cancer correlate with a better clinical outcome [50,51,52] and the experimental removal of Treg is only effective early after tumor inoculation [46]. Most likely, Treg suppress inflammation-dependent cancer, as well as the inflammation-dependent implantation of tumor cells, but promote non-inflammatory tumors by suppressing the inflammatory pathways necessary for their successful control.

### 2.3. Targeted IL-2 Assignment

**Figure 3:**
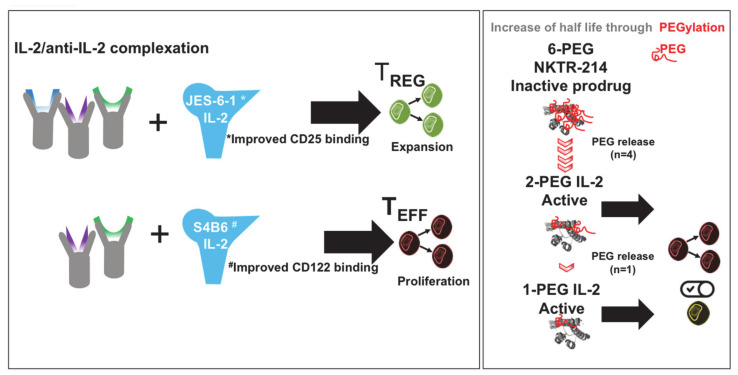
IL-2 modification for cellular targeting and prolonged half-life. IL-2 complexation with monoclonal antibodies (named JES-6-1 and S4B6) improves binding to either CD25 (JES-6-1/IL-2 complex) or CD122 (S4B6/IL-2 complex). This allows researchers to expand Treg numbers by JES-6-1/IL-2 complexes and increase proliferation of effector T cells by S4B6/IL-2 complexes. PEGylation of IL-2 forms 6-PEG IL-2, which is inactive and upon release of 4 PEG becomes activated. Further release of 1 PEG increases the half-life of active 1-PEG IL-2 in the circulation.

In order to be able to control the effect of therapeutically administered IL-2, attempts were being made to modify IL-2 so that it specifically binds low-affinity or high-affinity receptors. Using specific antibody clones, Jonathan Sprent and others showed that the blockage of individual IL-2 epitopes by antibodies has potential to target interaction of IL-2 to particular subtypes of receptors and, thus, cell types (Figure 3) [16,53,54]. This strategy, initially tested in mice, was being continued for human IL-2 [55]. Mutated forms of IL-2 were produced and clinically tested with the same goal, inter alia, by improving the binding to CD122 [56,57,58]. In addition, orthogonal IL-2 cytokine receptor pairs have been developed that interact with each other, but not with their natural cytokine and receptor counterparts, and show good efficacy in the mouse model [59]. While cytokine-antibody complexes can be much more effective than unmodified cytokines [54], the antibodies used must be humanized for clinical use.

An important disadvantage of natural IL-2 consists of its short life span. Here, the conjugation of therapeutic agents to polymeric carriers, such as polyethylene glycol (PEG), offers the advantage of improved drug solubilization and prolonged circulation [60]. Recently, PEGylated interleukin-2 (Bempegaldesleukin) has been shown to provide superior anti-tumor activity over native IL-2 and systemically expands anti-tumor CD8^+^ T cells while inducing Treg depletion in tumor tissue [61]. An extension of the half-life can also be achieved by using fusion proteins consisting of IL-2 and the crystallizable region (Fc) of the antibody fragment. The use of a mutated IL-2 that is not bound to CD25 results in long-lived IL-2-Fc variants in which the undesired Treg activation is avoided. Surprisingly, however, such variants are less effective than wild-type IL-2-Fc in mediating tumor rejection, since Fc-mediated immune effector functions appear to be required for Treg elimination [62].

### 2.4. IL-2-Decorated Nanoparticles to Boost Melanoma Immunity—Potential Advantages and Pitfalls

The production of IL-2 decorated particles for cancer therapy must follow the principles described here. Thus, the binding of IL-2 should be bound to the particles in such a way that preferential signaling via low-affinity IL-2 receptors is ensured. Alternatively, mutated IL-2 can be used that cannot bind to the high-affinity receptor. It is also conceivable to use antibodies (such as clone TCB1-3) against human IL-2 for particle- coupling that disfavors Treg stimulation [63].

The particles themselves must be stable, i.e., not subject to immediate disintegration or degradation, and must allow for extensive body distribution. Ideally, they should circulate in the blood and lymph for prolonged time and not accumulate locally. This means that they should be of a size and charge that is ignored by the mononuclear phagocytic (*MPS*) system [64] and made of a material that shows no accumulation in the liver [65]. Permanent, preferably covalent, binding must prevent IL-2 detachment in order to exclude uncontrolled effects (e.g., on Treg), which would otherwise lose the advantage of directional binding.

In principle, adjuvant stimulation of the immune system of cancer patients is associated with the risk of promoting autoimmunity [66,67]. Similarly, the effect and side effects of checkpoint inhibitors [68] show that anti-tumor immunity may not be achieved without this risk. Due to the similarity of tumor cells to their origin, effective tumor rejection by the immune system must even be understood as a local autoimmune reaction [69].

### 2.5. T Cell Activation through Delivery of Nanoparticle-Encapsulated IL-2

**Figure 4:**
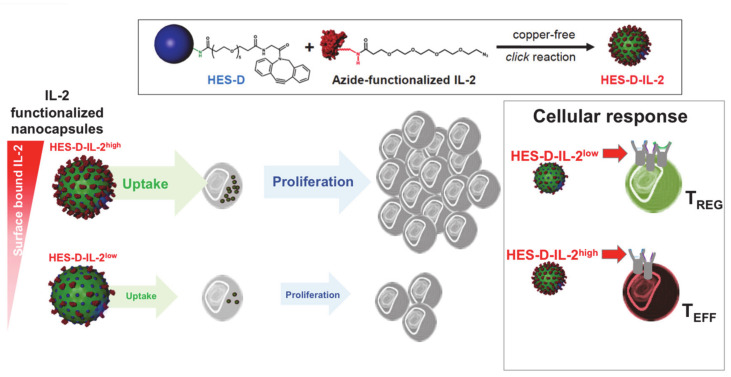
Bio-inspired nanoparticle-mediated IL-2 delivery strategies. Particle encapsulated IL-2 ensures a controlled release of the cytokine for treatment. Antibody-complexed IL-2 and dosed and oriented IL-2-coated particles enable receptor-specific targeting and activation.

A number of studies have investigated the effect of nanocarrier-encapsulated IL-2 on the immune system (Figure 4). For this purpose, IL-2 was loaded into liposomes [70], poly(milk-co-glycolic) (PGLA) particles [71,72], poly n-(2-hydroxypropyl)methacrylamide (HPMA) particles [73], Chitosan nanoparticles [74], or bundled carbon nanotubes [75]. In some of the studies, the nanocarriers were combined with other substances, including doxorubicin [74], TGF- α [71], a TGF-α inhibitor [70], or peptide-MHC complexes [76]. The aim of these investigations was mainly to improve immune responses against tumors by IL-2-induced activation of tumor-infiltrating T cells. In this context, Fadel et al. additionally attached antigens to bundled carbon nanotubes and combined this complex with polymer nanoparticles containing magnetite and IL-2. By using these complexes, a thousand times less IL-2 was required for T-cell activation and expansion compared to soluble IL-2, and a delay in tumor growth was achieved in a melanoma model.

As described above, FOXP3^+^ Treg displays a pivotal role in the control of autoimmune and inflammatory diseases and low dose of IL-2 has been shown to stimulate regulatory T cells and enhance their suppressive capacity [7,8]. Therefore, several studies have focused on nanocarrier-based IL-2 application to modulate regulatory T cell biology and to affect autoimmune phenomena. For this purpose, in an experimental setting, Horwitz et al. loaded PLGA nanoparticles with IL-2 and TGF-β and additionally coated them with anti-CD2/anti-CD4 antibodies to achieve T cell-specific targeting [71]. These nanoparticles induced an expansion of regulatory T cell in vitro and in vivo, and their systemic administration resulted in reduced disease activity in vivo in a murine model of Lupus erythematosus.

### 2.6. T Cell Targeting by IL-2-Functionalized Nanoparticles

**Figure 5:**
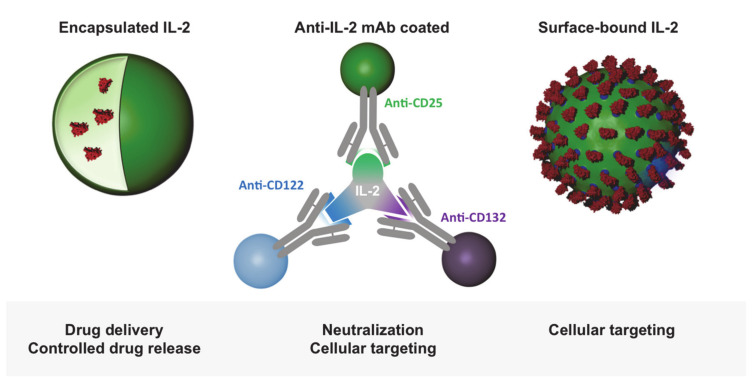
IL-2-functionalized nanocapsules for T cell targeting. Hydroxyethyl-starch (HES) nanocapsules were functionalized with dibenzylcyclooctyne (DBCO)-PEG_5_-NHS ester binding to NH_2_ groups on the carriers’ surface to introduce an active alkyne group (HES-D). IL-2 was azide-functionalized at its N terminus and coupled onto the DBCO-HES nanocapsules via copper-free *click* reaction. These capsules can be taken up by human and murine T cells. Different concentrations of IL-2 on the nanocapsule surface resulted in increased or reduced targeting and proliferation of Treg or effector T cells, respectively, (modified from Frick et al. [69]).

Alternatively to its release from particles, IL-2 can be used as a surface molecule on nanoparticles for direct stimulation (Figure 4). Wojta-Stremayr et al. generated antigen-presenting virus-like nanoparticles (VPN) that co-express IL-2 bound to different membrane anchors. They found that the fusion of the C-terminus of IL-2 with a minimal glycosylphosphtidylinositol anchor acceptor sequence with two intervening immunoglobulin-like domains of CD16b led to an optimal stimulation of T cells and induction of CD8^+^ T cell effector function in vivo [77]. Other studies reported the generation of IL-2 surface conjugates on PEGylated liposomes using covalently bonded succinimidyl-4-p-maleimidophenyl butyrate-modified IL-2 or an Fc scaffold fused to the C-terminus of the murine IL-2 [78,79]. Using an adoptive cell therapy model, Zheng et al. found that the vast majority of antigen-specific T cells reacted to IL-2-decorated liposomes in vivo after a single injection and that repeated administrations increased cytotoxic T cell activation and proliferation in melanoma-bearing mice. We functionalized nanocapsules with different amounts of IL-2 and studied how they interact with different T-cell populations (Figure 5) [80]. 

For this purpose, we coupled human IL-2, which interacts with both the human and (less strongly) the murine IL-2 receptors and, thus, can be studied with T cells of both species, to the surface of biodegradable hydroxyethyl starch (HES) nanocapsules. The hydroxy ethyl starch (HES) nanocapsules were synthesized by by an interfacial polymerization reaction in inverse miniemulsion [81]. This approach enables the simultaneous encapsulation of several drugs and reporter molecules in a nanocarrier in a high efficiency. Amino groups on the surface of the nanocapsules can be further functionalized with dibenzocyclooctyne (DBCO) groups for a 1,3-dipolar cycloaddition as copper-free click chemistry. The IL-2 has to be functionalized with azide groups at the N terminus of the protein. Subsequently, the modified IL-2 can be attached by 1,3-dipolar cycloaddition with the beforehand DBCO-functionalized HES nanocapsules, resulting in defined amounts of surface-bound IL-2 molecules on the nanocapsule surface.

HES-IL-2 nanocapsules exhibited a CD25-mediated uptake by CD25^+^ T cells confirmed by blockade with an anti-CD25 antibody basiliximab. Comparing the uptake by naïve CD25^-^, activated effector CD25^+^ and regulatory CD25^high^ human T cells revealed a very low incorporation of HES-IL-2 nanocapsules in naïve, and a moderate to high uptake by activated effector or regulatory T cells, respectively. Incubation with HES-IL-2 nanocapsules instigated CD4^+^ T cell proliferation upon uptake, confirming the requirement for IL-2/IL2R complex internalization in T cell proliferation (Figure 5) [82].

Reduction of HES-coupled IL-2 levels led to the nanocapsules interacting preferentially with CD25^high^ Treg. Most notably, the capsules were also significantly more strongly absorbed by CD4^+^CD25^+^ T cells in human T-cell or *peripheral blood mononuclear cell* (PBMC)-reconstituted immunodeficient RAG2^−/−^γc^−/−^ mice. We did not find any significant differences in the uptake by B cells, dendritic, and myeloid cells or macrophages, further confirming T cell specific targeting in vivo. Thus, our study clearly showed the development of biocompatible HES-IL-2 nanocapsules exhibiting the ability to target specific T cell populations with various IL-2 receptor affinities, in particular, regulatory T cells, through different amounts of surface-coupled IL-2 (Figure 5).

Besides a covalent binding of IL2 to the surface of nanocarriers, we could also successfully show an effective physical adsorption of IL-2 [83]. The IL-2 was adsorbed to HES nanocapsules at pH 6.1–7.5 and preserved its biological function. It is worth mentioning that the adsorbed IL-2 molecules did not desorb and were not exchanged over time by other proteins from human blood.

## 3. Conclusions

The immune-stimulatory cytokine IL-2 is a growth factor for T cells and natural killer cells. Considerable effort was invested in using IL-2 as therapeutic agent for a variety of diseases, ranging from autoimmune and inflammatory disorders, allograft rejection, to cancer. However, the adverse effects, in particular, IL-2 toxicity leading to the vascular leakage syndrome and the activation of effector and regulatory T cells depending on the dose of the cytokine, limited the use of IL-2 in the clinic. Therefore, different attempts were made to use the beneficial effects of the IL-2 pathway while limiting unwanted functions. These developments include numerous nanoparticle-based approaches to improve its therapeutic potential by control of IL-2 release, concentration, and targeting of specific T cell populations (effector/memory vs. regulatory T cells).
